# Simulating Carbon Stocks and Fluxes of an African Tropical Montane Forest with an Individual-Based Forest Model

**DOI:** 10.1371/journal.pone.0123300

**Published:** 2015-04-27

**Authors:** Rico Fischer, Andreas Ensslin, Gemma Rutten, Markus Fischer, David Schellenberger Costa, Michael Kleyer, Andreas Hemp, Sebastian Paulick, Andreas Huth

**Affiliations:** 1 Department of Ecological Modelling, Helmholtz Centre for Environmental Research–UFZ, Permoserstr. 15, 04318, Leipzig, Germany; 2 Institute of Plant Sciences, University of Bern, Altenbergrain 21, CH-3013, Bern, Switzerland; 3 Department of Biology and Environmental Sciences, University of Oldenburg, Carl-von-Ossietzky–Strasse 9–11, 26111, Oldenburg, Germany; 4 Dept. of Plant Systematics, University of Bayreuth, Universitaetsstr. 30-31, 95440, Bayreuth, Germany; 5 Institute of Environmental Systems Research, University of Osnabrueck, Barbarastraße 12, 49076, Osnabrueck, Germany; Vrije Universiteit, NETHERLANDS

## Abstract

Tropical forests are carbon-dense and highly productive ecosystems. Consequently, they play an important role in the global carbon cycle. In the present study we used an individual-based forest model (FORMIND) to analyze the carbon balances of a tropical forest. The main processes of this model are tree growth, mortality, regeneration, and competition. Model parameters were calibrated using forest inventory data from a tropical forest at Mt. Kilimanjaro. The simulation results showed that the model successfully reproduces important characteristics of tropical forests (aboveground biomass, stem size distribution and leaf area index). The estimated aboveground biomass (385 t/ha) is comparable to biomass values in the Amazon and other tropical forests in Africa. The simulated forest reveals a gross primary production of 24 t_c_ha^-1^yr^-1^. Modeling above- and belowground carbon stocks, we analyzed the carbon balance of the investigated tropical forest. The simulated carbon balance of this old-growth forest is zero on average. This study provides an example of how forest models can be used in combination with forest inventory data to investigate forest structure and local carbon balances.

## Introduction

Tropical forests have long been recognized as highly productive ecosystems [1]. As such, these forests play a key role in the global carbon cycle. More than 50% of global carbon stored in living biomass is located in the tropics [2]. Globally, these forests are considered as a carbon sink with 1.2 Gt C per year [2–4]. On a local scale, however, whether tropical forests serve as carbon sinks or sources is unclear due to their heterogeneity and the potential impacts of climate change and land use [5,6]. While most studies of tropical forests concentrate on regions in South America or Asia (e.g., Amazonas: [7,8,9,10,11]), African tropical forests have been somewhat neglected, despite the fact that they have the highest deforestation rates worldwide (yearly 1.01% in East Africa, [12]). In Africa, large areas of tropical forest are located in the Congo basin, in the Eastern Arc Mountains and in the Kilimanjaro region [13].

Mt. Kilimanjaro supports high biodiversity due to its unique range of vegetation zones and climatic conditions. Savannas and different forest types can be found along the elevation gradient [14]. Before human settlement began here over 2,000 years ago, the southern elevation belt between 1,000 m and 1,800 m was covered by tropical lower submontane forest. Today, to a large extent these forests have been converted into agroforestry systems, grasslands or coffee plantations. Other natural ecosystems at Mt. Kilimanjaro are strongly impacted by burning or logging, but they are also subject to climate change [14–19]. This has consequences for forest functions such as the allocation of biomass and carbon, water storage, climate regulation, and the provision of food [20]. Recent field surveys and forest inventories have been used to monitor forest structure and carbon stocks (e.g., [21,22,23]) under ongoing land use and climate change. However, these surveys provided only a snapshot in time of the forest and did not allow conclusions to be drawn about forest dynamics and forest carbon emissions. In addition, forest inventories are spatially limited and represent a sample of a large ecosystem. In general, analyzing the heterogeneous structure and dynamics of tropical forests on a local scale poses a challenge. Here, forest models play an important role in process understanding and for a projection to larger scales [24,25]. Process-based forest models simulate forest processes such as tree growth, mortality, and regeneration. For our study we used the local process-based forest model FORMIND [24,26–28] in combination with forest inventory data. This model simulates the dynamics of tropical forests over several hectares and centuries. FORMIND uses the concept of plant functional types (PFT) to comply with the high number of tree species found in the tropics.

The forest model FORMIND was applied here to reproduce the patterns of an almost undisturbed tropical forest at Mt. Kilimanjaro. Specifically, we assessed (1) the patterns in number of trees, basal area, aboveground biomass, stem size distribution, and leaf area index. (2) We calculated a local carbon balance of the investigated forest. (3) We asked if this lower montane forest is a carbon sink or source.

## Materials and Methods

### Study Site

At Mt. Kilimanjaro the submontane and lower montane forest belt between 1,000 m a.s.l. and 1,800 m a.s.l. has to a large extent been converted to small-scale coffee-banana plantations, the so-called “Chagga home gardens”, a special type of agroforestry. The maximum population density in the area is 1,000 persons per km^2^ [29]. The tropical submontane forest investigated in the present study is located in a steep valley and is largely undisturbed due to restricted accessibility. In this area five different forest research sites were established with a total size of two hectare. An extensive forest inventory was carried out in 2012 by the German Research Foundation (DFG) within the Research Unit FOR1246 (Kilimanjaro ecosystems under global changes: linking biodiversity, biotic interactions and biogeochemical ecosystem processes; www.kilimanjaro.biozentrum.uni-wuerzburg.de) in cooperation with the Kilimanjaro National Park (http://www.tanzaniaparks.com/kili.html). Andreas Hemp, Markus Fischer and Ingolf Steffan-Dewenter are Spokesperson of the DFG project and are responsible for the permits for the investigated forest at Mt. Kilimanjaro. This study was not carried out on private land and we confirm that the field studies did not involve endangered or protected species.

**Fig 1:**
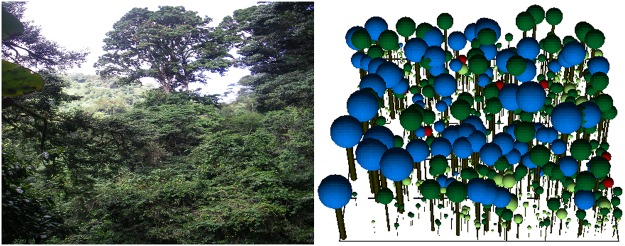
The study site. Left: Photograph of the study site at Mt. Kilimanjaro (FLM3). Right: Visualization of the forest simulated with FORMIND, showing all trees with a stem diameter > 5 cm for a forest area of 1 ha. The colors of the crowns represent the six plant functional types (PFTs).

Concerning this forest inventory, for each tree with DBH > 10 cm the specific position, stem diameter, tree height, crown expansion, and species identity were determined. The forest inventory of all five research sites were used to parameterize the model. These forest research sites distinguish from each other by forest structure and species composition mainly due to the spatial location and disturbance history. For testing the simulation outcome we used a nearly undisturbed research site (forest lower montane, denotation FLM3, 50m x 50m, S3.260150°, E37.417458°, see Fig 1). The dataset of this forest stand resembles an equilibrium situation which could be confirmed by the tree species found in this site (dominated by late succession species; fraction of pioneer trees is less than 1%). This test study site is dominated by *Heinsenia diervilleoides* (36% of all trees) and *Strombosia scheffleri* (24%), *Entandophrangma excelsum* (6%) and *Garcinia tansaniensis* (5%). In total, we discovered 93 trees and 16 tree species at the test study site. The observations of the study site were linear scaled up to one hectare to be comparable to our simulation results of the forest model (1 ha forest simulations).

### Overview of the FORMIND forest model

Forest gap models are tools for investigating forest dynamics [30–32]. These models simulate the succession of forests. Considering a forest gap caused by a falling tree, for instance, the opened canopy results in increased light availability and hence to the growth of sub-canopy trees and recruits [33,34]. The FORMIND forest gap model [24,26] is an individual- and process-based simulation model designed specifically for tropical forests that considers the complex age structure of the tree community. FORMIND has been extensively tested and applied to tropical forests in Panama, Malaysia, French Guyana, Venezuela, Mexico, Brazil, Madagascar and Paraguay [26–28,35–42], and even to European forests [43] and grasslands [44].

By using this individual-based model, every single tree is simulated. The model considers four main process groups: growth of single trees (increment of tree biomass), mortality, recruitment, and competition for light and space. The simulated forest area was divided into patches according to the typical size of tree fall gaps (20 m x 20 m). The vertical leaf distribution and light availability was calculated for each patch. The biomass growth of each tree was determined on the basis of a carbon balance including photosynthesis, respiration, biomass allocation, and litter fall. Photosynthetic production in the canopy depends on local light availability. Forests of up to 1,000 hectares can be simulated over a time period of several centuries. In this study the FORMIND model was used to investigate the biomass production and mortality of the forest ecosystem. In addition, above- and belowground carbon stocks and the resulting net ecosystem exchange (NEE in t_c_ha^-1^yr^-1^) was calculated as the balance of carbon sequestration and carbon emissions (see S1 Text). The forest ecosystem is considered to be a carbon sink if it absorbs more carbon then it releases (NEE is positive). If, on the other hand, the heterotrophic respiration is greater than the net primary production, the forest is a carbon source (NEE is negative). A detailed description of the FORMIND model can be found in S1 Text or at www.formind.org.

### Species grouping

Grouping tree species into plant functional types (PFT) is a well-established classification method in vegetation ecology [45,46]. For the purpose of this study, local tree species with similar trait expressions were assigned to one of six groups according to their maximum height and light requirements (see Table 1, S2 Text for the full species list and their traits). We used three height classes (<16m, 16–33m, >33m) and three classes of shade tolerance (shade-tolerant—climax species, shade-intolerant—pioneer species, and intermediate shade-tolerant species). The grouping of tree species into shade tolerance classes was based on measured plant functional traits and expert knowledge. Specifically, we used stem dry matter content and leaf nitrogen content to assign species to a shade tolerance scale (see S2 Text).

**Table 1:** Species grouping into six plant functional types (PFT) for the investigated forest.

PFT	Maximum Height (m)	Light Class	Exemplary Tree Species
1	>33	Shade tolerant	*Strombosia scheffleri*
2	16>33	Shade tolerant	*Heinsenia diervilleoides*
3	16>33	Intermediate	*Ficus sur*
4	16>33	Shade intolerant	*Polyscias albersiana*
5	<16	Shade tolerant	*Leptonychia usambarensis*
6	<16	Shade intolerant	*Cyathea*.*manniana*

The grouping consists of three size classes and three light demand classes. The size class depends on the maximum attainable height of the respective tree species. The light class indicates shade tolerance: shade-tolerant trees (climax species), shade-intolerant trees (mainly pioneer species) and intermediate shade-tolerant trees.

### Simulation Experiments

We analyzed forest succession on 1 hectare over 1000 years, starting with bare ground conditions and without climate limitations. To assess the variability in the forest model we had 10 repeated simulation runs. Two types of analysis are presented: First, for a comparison of the model output with field data, we calculated the mean of simulated forest attributes over the last 300–1,000 years, based on the assumption that the forest is in the equilibrium state for the entire period. In particular, we analyzed aboveground biomass, basal area, stem numbers (dbh>10 cm), stem size distribution, and leaf area index (LAI). Second, we analyzed forest attributes which are difficult and costly to measure in a real forest, namely gross primary production (GPP), aboveground respiration of the vegetation, the resulting net primary production (NPP), and mortality. Additionally, we calculated above- and belowground carbon stocks and net ecosystem exchange (NEE). Details of the forest model and all parameter values are given in S1 Text.

### Calibration Process

For this study, most of the FORMIND model parameter values were determined from the local forest inventory (e.g., allometric relationship between DBH and tree height), while some parameter values were taken from the literature (see S1 Text). Parameter values concerning tree growth and mortality are unknown due to the lack of repeated forest surveys. These parameters were estimated by a calibration process, comparing the simulated mature forest with the measured forest inventory from the test site. In detail, we compared the total aboveground biomass and the fraction of biomass for each PFT for all trees with a DBH > 10 cm. The simulated biomass was averaged over the last 300–1,000 years. Aboveground biomass of the field data was calculated by summing up the biomass of the individual trees using the same assumptions as those used in the FORMIND model [24]. For this calibration process we varied growth and mortality parameter values and calculated the mean square error between field data and the averaged equilibrium state of the corresponding simulated value [47]. We used inverse parameterization techniques to find an optimal parameter set [48,49]. For the optimization 50,000 different simulation runs were analyzed. All parameter values can be found in S1 Text. To validate the forest model we compared the simulated basal area, stem size distribution and leaf area index with the corresponding observations in the research test site (FLM 3, see S1 Dataset).

## Results

**Fig 2:**
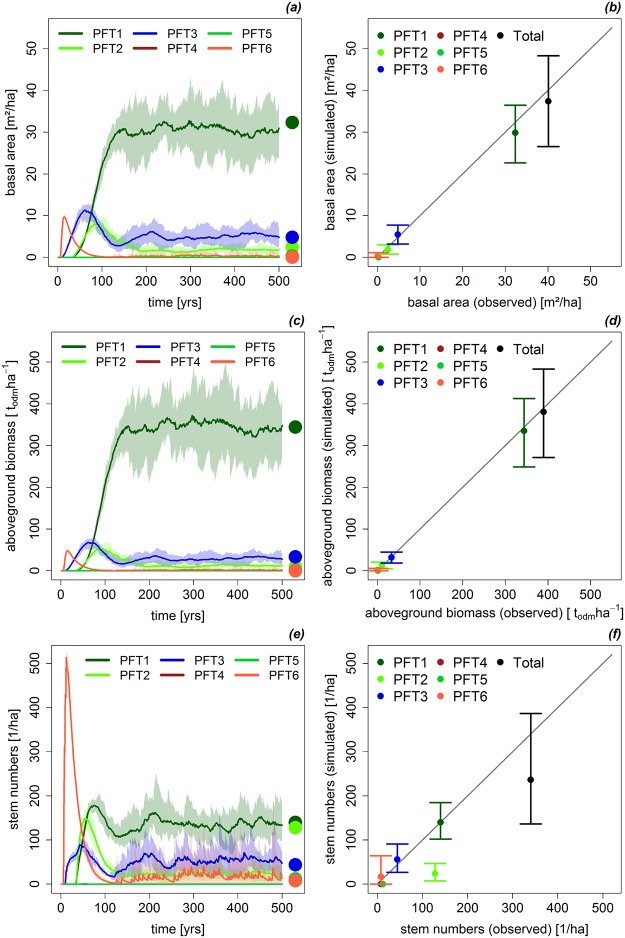
Simulation vs. Measurements. Comparison between simulated variables and measurements from the forest inventory at Mt. Kilimanjaro. Presented are simulated basal area (a), aboveground biomass (c), and stem numbers (e) over time for each PFT. The points represent the measured value from the inventory data. A direct comparison between mean values of the simulation (300–1000 years) versus measured values for each PFT is also presented. The vertical lines represent the variation of the simulated value for basal area (b), aboveground biomass (d), and stem numbers (f).

To test the developed parameterization for the forest at Mt. Kilimanjaro we compared simulated aboveground biomass, basal area and stem numbers with the field data for the test study site (scaled to one hectare). The comparison is made on the level of plant functional types (PFT). We found that shade-tolerant (PFT1&2) and intermediate shade-tolerant (PFT3) tree species are most dominant in the investigated forest. Especially tall-growing tree species corresponding to PFT1 dominate the study site. The simulated forest reproduced this high PFT1 fraction (see Fig 2) for all investigated attributes (basal area, aboveground biomass, stem number).

The aboveground biomass of the simulated forest (385 t_odm_/ha) was 2% higher than observed in the field data (Fig 2c/2d). The aboveground biomass of all PFTs was reproduced well by the model (difference ±4%). This is due to the fact that the biomass estimations of the study site were used to calibrate the forest model. PFT1 is the dominating PFT in terms of biomass with 330 t_odm_/ha. The high variation in the total aboveground biomass (see Fig 2c/2d) is due to the death of tall trees of PFT1. The biomass of PFT1 fluctuated between 250 and 450 t_odm_/ha over time.

The total basal area of the simulated data (39 m^2^/ha) was close to the observed field data (40 m^2^/ha, see Fig 2a/2b). The basal area of PFT1 was slightly underestimated (5%) in the simulation. Simulated basal areas of the remaining PFTs reproduced the field data very accurate (difference ±2%). The consistence of simulated and observed basal area is due to the fact that biomass values, which by definition are closely linked to basal area, were used for calibration of the model. The mean total simulated basal area was 39 m^2^/ha and fluctuated between 28 and 49 m^2^/ha over time. This variation was mainly due to fluctuations in the number of tall-growing shade-tolerant canopy trees (PFT1).

Total stem numbers were 30% lower in the simulation (240 trees per hectare) than in the field inventory (350 trees per hectare; see Fig 2e/2f). This difference is caused by PFT2 (shade-tolerant, up to 33 m in height), which had 100 trees per hectare less in the simulation than observed in the field. Overall, the stem numbers varied over time due to stochastic processes in the model (between 150 and 400 trees per hectare).

**Fig 3:**
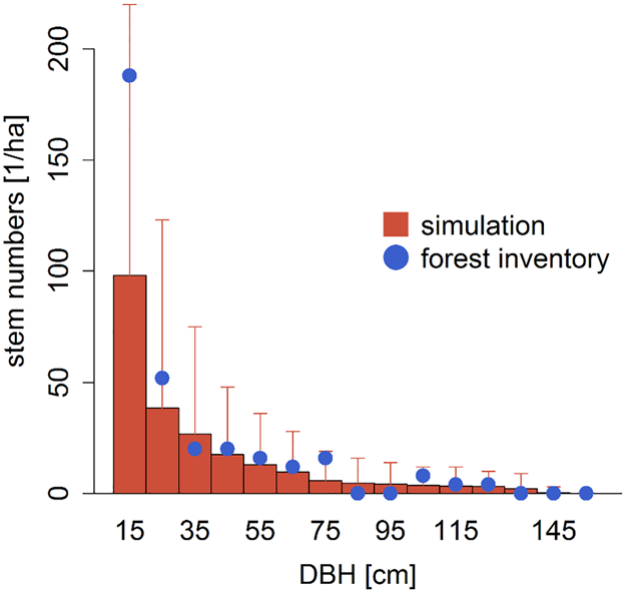
Simulated stem size distribution. Number of trees for different DBH—classes. The red bars indicate the simulated stem size distribution with its variation. The blue line indicates the measured stem size distribution for the investigated forest at Mt. Kilimanjaro.

The stem size distribution of the simulated data was similar to the field data for all trees with a DBH > 20 cm (Fig 3). The number of trees between 10 and 20 cm DBH was underestimated up to 50% compared to the field data. Overall, the difference in simulated versus observed stem numbers is mainly due to fewer small trees within PFT2 with a DBH between 10 cm and 20 cm (shade-tolerant tree species).

**Fig 4:**
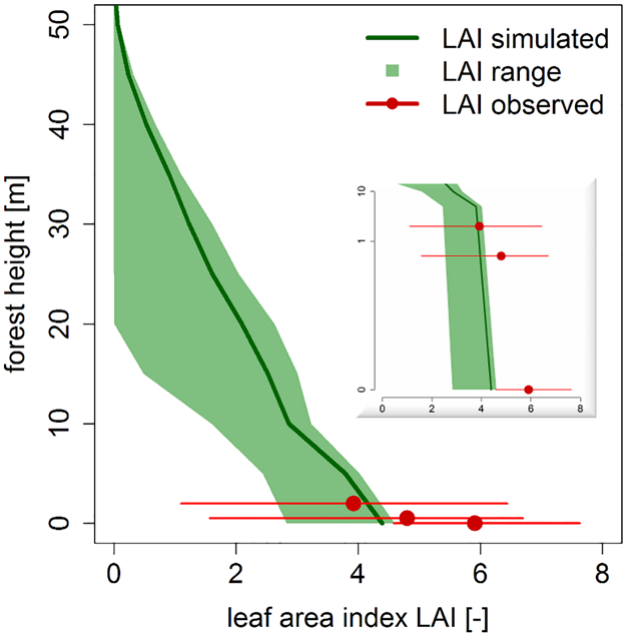
Leaf area index (LAI) for different height layers in a forest. The green line is the mean of the simulated LAI, the green area is the range of the LAI in different height layers. The red points are the mean measurements of the LAI in the investigated forest, the red lines are the measured range.

The leaf area index (LAI) was repeatedly measured at different heights (0 m, 0.5 m, 2.0 m) and ranged from 6 at ground level to 4 at 2.0 m above ground. The LAI in the simulated forest at 2.0 m height was similar to the observed values (see Fig 4). However, at ground level the simulated LAI was 20% lower than that observed in the field. This is due to the fact that the simulation has 30% fewer trees compared to the real forest. Additionally, this could be due to small plants growing in the understory in the field (shrubs, herbs) which reduce the light at ground level but was not taken into account in the simulation. There was a high degree of LAI variation in the simulated forest, which is also typical for real forests in the investigated area due to the complex forest structure [23].

**Fig 5:**
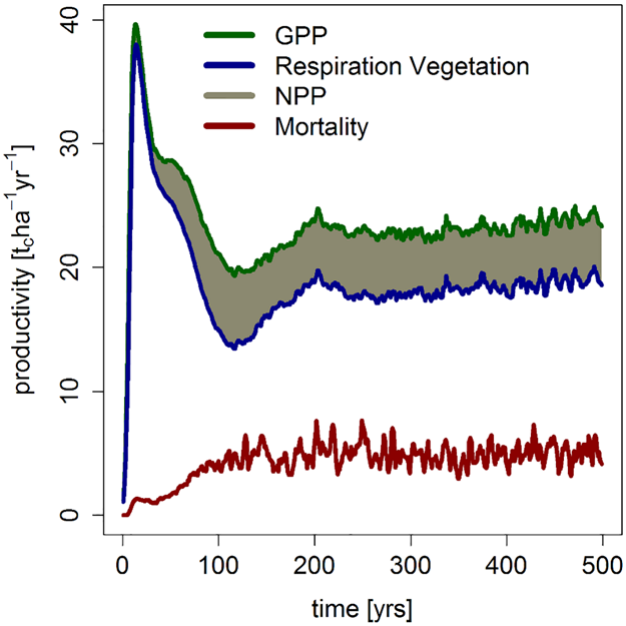
Simulated productivity and tree mortality over time. Presented are gross primary production (GPP), respiration of the vegetation, and the resulting net primary production (NPP). The red line indicates the loss of biomass due to mortality events.

In the simulated mature forest, gross primary production (GPP) reached 23.5 t_c_ha^-1^yr^-1^ (see Fig 5). Respiration of the aboveground vegetation contributed 18.8 t_c_ha^-1^yr^-1^. Thus, mean net primary productivity (NPP) was approximately 4.7 t_c_ha^-1^yr^-1^. The total loss of aboveground biomass due to mortality events was in the same magnitude as NPP (4.7 t_c_ha^-1^yr^-1^), which is typical for forests in the late successional phase [50,51].

**Fig 6:**
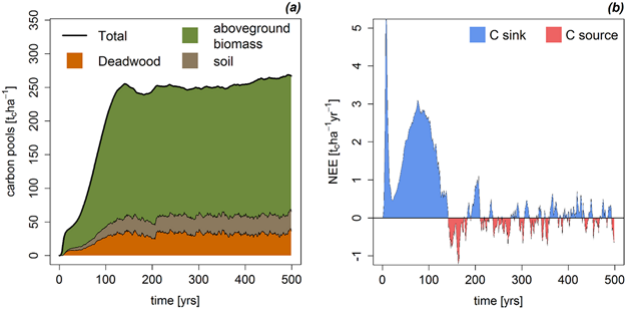
Carbon stocks and carbon fluxes. Simulated forest carbon pools and net ecosystem exchange (NEE) over time. (a) Presented is the amount of carbon in the living biomass, in the deadwood, and in the soil. (b) The NEE is a measurement of how much carbon is entering and leaving the forest. The simulated NEE started with a forest clearcut. Positive values indicate a carbon sink.

To analyze the carbon stocks, we distinguished between three different carbon pools: carbon stored in the living vegetation, carbon in the deadwood and carbon stored in the upper soil layer. According to the simulation, the mature forest stored 193 t_c_/ha in the living vegetation, 32 t_c_/ha in the deadwood and 28 t_c_/ha in the soil (see Fig 6a). In total, there was 253 t_c_/ha carbon stored above- and belowground in the investigated tropical forest.

Using the simulated carbon stocks and the fluxes between these stocks it is possible to estimate the carbon balance. According to the model results, the forest was a strong carbon sink during the first 150 years of simulation (see Fig 6b). During this phase the forest stored up to 6 t_c_ha^-1^yr^-1^. After about 200 years of simulation the NEE got balanced with values between +1.0 and -1.0 t_c_ha^-1^yr^-1^. In the mature state, the forest is in a nearly carbon neutral state.

## Discussion

The region around Mt. Kilimanjaro represents a biodiversity hotspot because of its unique range of vegetation zones and climatic conditions [17]. We parameterized the FORMIND forest model for a lower montane tropical forest at Mt. Kilimanjaro. The simulation results showed that the model successfully reproduces measured basal area and aboveground biomass due to the fact that observed biomass values were used to calibrate the model. Total stem numbers are underestimated due to fewer small trees (understory) in the simulated forest (compared to the field data). However, we assume that this underestimation in stem number is of minor importance in the carbon dynamic analysis because most carbon is stored in huge trees. To validate the model simulations we used independent measurements of leaf area index which could be reproduced very well by the forest model. The simulated aboveground biomass (385 t/ha) is comparable to biomass estimations in the Amazon (312–464 t/ha; [9]) and lowland tropical forests in Africa (350–490 t/ha; [13,52]). Overall, we conclude that the FORMIND forest model is an appropriate tool for reproducing main structural characteristics of tropical lower montane forests at Mt. Kilimanjaro.

Tropical forests store a large amount of carbon [2,53]. However, further efforts have to be made to measure the productivity and net ecosystem exchange of tropical forests in the field [3,54–56]. The average carbon stocks of African old-growth tropical forests were found to increase by one ton of carbon per hectare per year, as estimated by Lewis *et al*. based on monitoring of 79 forest inventory plots [52]. This might be due the positive effect of CO_2_-fertilization on biomass production in tropical rainforests [52]. An alternative explanation could be that the forests investigated by Lewis may not have been in an old-growth phase and therefore had a positive carbon balance [57]. On a local scale, our study cannot confirm the finding that African tropical forests are carbon sinks. Under our assumptions of constant climatic conditions and natural disturbances only (falling of large dying trees), the simulated carbon balance of an old-growth forest is zero on average. Grant *et al*. also found a balanced NEE in tropical forests 100 years after a clear-cut [8]. However, a changing climate could result in different NEE values for the investigated region. This possibility needs to be investigated in future studies.

The forest model used here includes a detailed description of important forest processes (tree growth, mortality, recruitment, competition). Uncertain parameters were calibrated using forest inventory data. However, parameters related to growth processes are difficult to estimate and repeated forest inventories would be necessary [58]. As no such repeated inventories were available, we parameterized the growth processes of the model with established data from the literature, expert knowledge, and inverse parameterization techniques. All model parameters were checked and are in the typical range for tropical forests [24,59,60]. One additional uncertainty results from the limited size of the forest test site (50m x 50m). We scaled up the observations from the forest inventory to one hectare to get the same scale than the forest model simulations. For this upscaling a linear approach was used which assumes that the whole investigated forest is in the same successional stage than the inventory of the study site (old-growth, late successional stage). However, in a real forest a mixture of successional stages can be found due to tree falling and gap building. For improved upscaling methods more information about the amount of forested area in different successional stages and the corresponding tree size distribution are needed. A forest inventory of several hectares would increase the confidence level of the parameter values and the resulting forest simulations [61,62].

To conclude, this study shows that process-based forest models can be used to investigate the carbon balance of tropical forests on a local scale. Aboveground biomass and net ecosystem exchange are especially important for the analysis under different conditions. It would be interesting to investigate forest dynamics in the context of climate change (less precipitation, higher temperatures) and land use (logging, burning) in future studies. Using FORMIND, disturbances can be simulated in a straightforward manner because this model is individual-based (e.g., tree harvesting; [26,36]). This approach makes it simple to select single trees and remove them from the simulated forest. Furthermore, the effects of changes in precipitation regimes can be analyzed by implementing a soil water module and including the relationship between tree productivity and soil water content [41,63]. FORMIND can also be used to explore the impact of temperature changes once functions are available to determine how tree productivity and tree respiration depend on temperature in tropical regions [64]. Finally, the simulation of tropical forests on a local scale could enhance remote sensing analysis [65], e.g. the relationship between forest structure and aboveground biomass under different climate scenarios or disturbance regimes.

## Supporting Information

S1 Dataset Comparison between FORMIND simulations and field observations. (XLSX)

S1 Text Detailed FORMIND description including all parameters. (PDF)

S2 Text Species grouping into plant functional types by species traits. (DOCX)
