# Supplementary material for: Simulating Carbon Stocks and Fluxes of an African Tropical Montane Forest with an Individual-Based Forest Model
Source: PLoS One. 2015 Apr 27;10(4):e0123300. doi: 10.1371/journal.pone.0123300 (PMC4410999; doi:10.1371/journal.pone.0123300)
Supplement: S1 Text — (PDF) [file pone.0123300.s002.pdf]

A General concept

FORMIND 3.0 is an individual-based, spatially explicit and process-based model designed for simulating species-rich vegetation communities. This document introduces only a specific version of FORMIND 3.0 (SVN-Built 1619) which simulates forest dynamics at Mt. Kilimanjaro, Tanzania. For a full model description of FORMIND 3.0 please go to www.formind.org. The full description shows the entire range of different model versions, which can be currently applied (i.e. the choices of different geometries of the vegetation, of the climatic zone or of various disturbance events).

In FORMIND 3.0 vegetation is simulated on an area of size A_{area}, which is a composite of regularly ordered, quadratic patches of size A_{patch} [m^2] uniquely described by their location within the area (Fig. 1). Individual trees grow within the patches, but do not have spatially explicit positions within a patch.

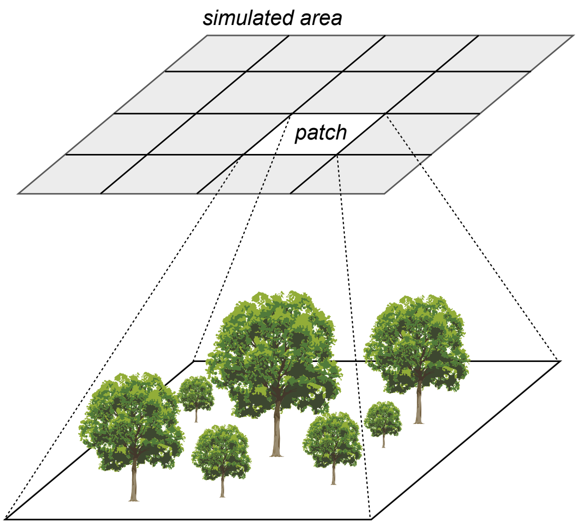

Figure 1: *Illustration of the simulated area and its composition of regularly ordered patches. Individual trees do not have spatially explicit positions within the patches. Only for an illustrative purpose, we show positioned trees on an exemplary patch.*

The trees change their size during the simulation according to a type-specific set of eco-physiological and morphological parameters used within the modelled processes. The modelled processes are simulated on different levels: (i) area-level, (ii) patch-level or (iii) on the level of a single tree .

Within each time step t_y, the following main processes are calculated:

- **Chapter C - Recruitment and establishment**
  Establishment of recruited seeds is modelled on the patch-level, whereby the recruitment of new trees is simulated on the area-level.

- **Chapter D - Mortality**
  First, an event-driven mortality due to crowding can take place on the patch-level. Afterwards, mortality affects each trees including the chance of a dying tree to fall down and damage other trees

- **Chapter F - Growth**
  The growth of a single tree is determined by its gross productivity, respiration and type-specific morphological parameters. Respiration is calculated on the level of an individual. An increase in biomass per tree is modelled as the difference between gross photosynthesis and respiration. The allocation of the resulting biomass increase (including the increase of geometrical properties according to chapter B) act on the level of a tree .

The modelled processes, which are summarized within the above mentioned main processes, are scheduled in a serial way. For details on the modelled processes and their schedule see Fig. 2.

Periodic boundary conditions are used. That means processes leaving one side of the simulation area are entering the area on the opposite side again.

For the purpose of calculations within the processes of light climate and crowding mortality, the above-ground space is discretized into vertical height layers of constant width Δh. Table 1 shows general input parameters.

Table 1: *General and technical parameters.*

Name	Symbol	Value	Unit	Reference
Simulation time	t_{end}	4500	year	-
Time step	t_y	1	year	-
Simulation area	A_{area}	25	hectare	-
Patch area	A_{patch}	400	m^2	-
Number PFTs	$MaxGrp$	16	-	-
Width of height layers	Δh	0.5	m	-

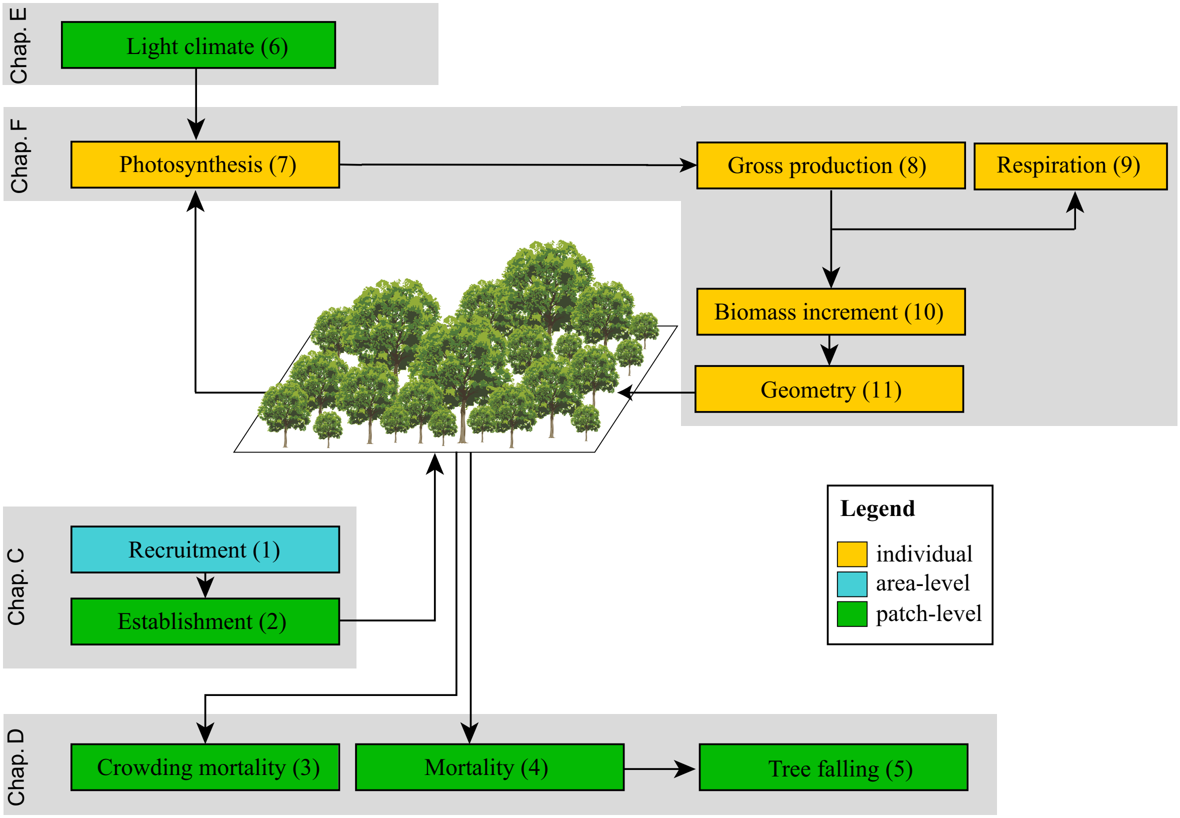

Figure 2: *Block diagram of the modelled processes. Different colours indicate the spatial scale on which each process is calculated (blue = area, green = patch, orange= individual). Italic written boxes show processes which are simulated with time steps of higher resolution than* t_y*. Numbers in brackets within each box show the serial order of their calculation within one time step* t_y*. Grey frames that underly these boxes group them according to the above mentioned main processes and their corresponding chapters.*

B Geometry

Although individual trees in real forests should not have necessarily identical shapes, we model each tree by a cylindrical stem and a cylindrical crown (Fig. 3). The geometry of an individual can be described completely by the following size characteristics: stem diameter (D), height (H), crown diameter (C_D), crown length (C_L) and crown projection area (C_A) as shown in Fig. 3.

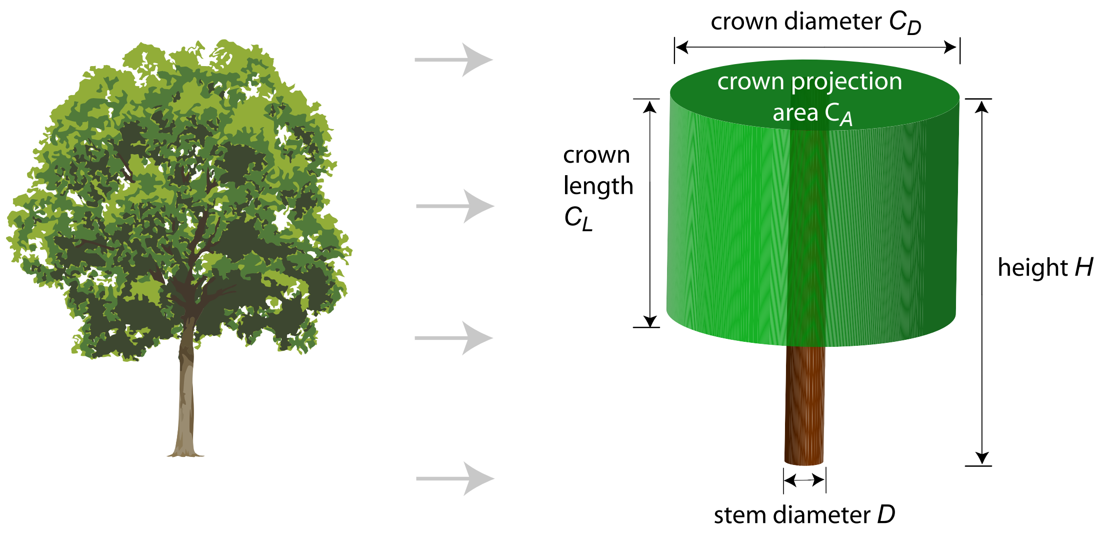

Figure 3: *Geometrical representation of a single tree . The following abbreviations describe size characteristics of the modelled tree geometry: D - stem diameter, H - height, C_D - crown diameter, C_L - crown length, C_A crown projection area.*

These size characteristics are functionally related to each other. In the following, we describe the functional relationships used. Parameters of the described relationships can vary between different tree types.

B.1 Height - Stem Diameter - Relationship

The height H [m] of a tree relates to its stem diameter D [cm] by:

$$H = h_0 \cdot D^{h_1}, \tag{1}$$

where h_0 and h_1 are type-specific parameters.

B.2 Crown length - Height - Relationship

The crown length C_L [m] of a tree is modelled as a fraction of its height H [m]:

$$C_L = c_{l0} \cdot H, \tag{2}$$

where c_{l0} is a type-specific parameter.

B.3 Crown diameter - Stem diameter - Relationship

The second dimension of the cylindrical crown, i.e. the crown diameter C_D [m] of a tree relates to its stem diameter D [cm] by:

$$C_D = c_{d0} \cdot D^{c_{d1}} - c_{d2}, \tag{3}$$

where c_{d0}, c_{d1} and c_{d2} are type-specific parameters.

B.4 Crown area - Crown diameter - Relationship

The crown projection area C_A [m^2] of a tree is simply the ground area of the modelled cylindrical crown:

$$C_A = \frac{\pi}{4} \cdot C_D^2. \tag{4}$$

B.5 Aboveground biomass - Stem diameter - Relationship

The aboveground volume of a tree captures biomass (i.e. organic dry matter). Aboveground biomass B [t_{ODM}] of a tree is calculated in relation to its stem diameter D [m] and height H [m] by:

$$B = \frac{\pi}{4} \cdot D^2 \cdot H \cdot f \cdot \frac{\rho}{\sigma}, \tag{5}$$

whereby the calculation simply represents the volume of the tree stem (according to its geometry) multiplied by three factors, which describe the biomass content more concisely.

Firstly, f [-] denotes a type-specific form factor, which accounts for deviations of the stem from a cylindrical shape. Secondly, the parameter ρ in [t_{ODM}/m^3] represents the wood density, which describes how much organic dry matter per unit of volume the stem contains. Thirdly, the division by the parameter σ [t_{ODM}/t_{ODM}], which represents the fraction of total aboveground biomass attributed to the stem, results then in the total aboveground biomass B.

In contrast to the constant parameters ρ and σ, the form factor f [-] can change during the growth of an individual with respect to its stem diameter D [cm] :

$$f = f_0 \cdot D^{f_1}, \tag{6}$$

whereby f_0 and f_1 are type-specific parameters.

B.6 Leaf area index - Stem diameter - Relationship

In general, aboveground biomass is divided between woody biomass captured in the stem and green biomass captured in the crown leaves. Important for the photosynthetic production of a tree is the green biomass captured in crown leaves. As leaves absorb radiation for photosynthesis, the total amount of one-sided leaf area per unit of crown projection area (i.e. the individual's leaf area index) is of main interest. The leaf area index LAI [m^2/m^2] of a tree relates functionally to its stem diameter D [cm] by:

$$LAI = l_0 \cdot D^{l_1}, \tag{7}$$

whereby l_0 and l_1 are type-specific parameters.

All parameters mentioned above are listed in Tab.2.

B.7 Maximum Values

The trees cannot grow indefinitely in FORMIND 3.0 . Therefore, we introduce the following maximum values for a plausible geometry of a mature individual:

- maximum stem diameter D_{max} [m]
- maximum height H_{max} [m]
- maximum biomass B_{max} [t_{ODM}]

Either the maximum stem diameter or the maximum height is given as a type-specific input parameter. Those two maximum values, which are not predefined, are then derived using the functional relationships mentioned in section B.1 and section B.5. The maximum values are used in section F. In this study maximum height is predefined for each plant functional type (see Table 2).

Table 2: *Summary of the type-specific geometrical parameters for all plant functional types (PFT).*
References: [1]*field data* [2]*Dislich et al., 2009* [3]*Nenninger, 2006* [4]*Rüger et al., 2007*

Symbol	PFT 1	PFT 2	PFT 3	PFT 4	PFT 5	PFT 6	
$H_{\max}$	56	33	33	28	16	16	[1]
h_0	3.28	4.64	4.82	4.27	4.35	3.00	[1]
h_1	0.57	0.41	0.44	0.43	0.34	0.60	[1]
c_{l0}			0.30				[2,4]
c_{d0}			0.60				[1]
c_{d1}			0.68				[1]
c_{d2}			0.00				[1]
ρ	0.55	0.54	0.41	0.40	0.52	0.47	[1]
σ			0.70				[2,3]
f_0			0.77				[2]
f_1			-0.18				[2]
l_0			2.00				[2]
l_1			0.10				[2]

C Recruitment and Establishment

C.1 Global in-growth rates

The number of recruited seeds is assumed to be brought into the local community from an intact forest community surrounding the simulated area. This number N_{seed} [$^1/_{\text{yr ha}}$] is thereby a constant type-specific parameter independent of the density of individuals already existing on the simulated area.

The recruited seeds directly enter the seed pool, but they may only germinate and establish in the next time step. Each patch is assigned an own seed pool. The recruited seeds are distributed uniformly across the patches and added to the corresponding seed pool in an amount of:

$$N_{pool} = \left\lfloor \frac{N_{seed}}{\#patches} \right\rfloor . \tag{8}$$

If the number of ingrowing seeds N_{seed} is not a multiple of the number of patches $\#patches$, a certain number of seeds will remain which are distributed randomly to the patches. For this, the patches are considered one by one incrementally starting with the first. Within each considered patch and for each remaining seed, which has not been distributed yet, its probability of assignment to the currently considered patch is compared with a random number (uniformly distributed in [0;1]). In the case of successful assignment (i.e. random number $\leq 1/\#patches$), the seed number per patch N_{pool} is incremented and the number of remaining seeds decremented. At the end, the last patch receives all remaining seeds.

Before the start of the simulation, N_{init} seeds already existing in the seed pool per patch (i.e. $N_{pool} = N_{init}$) can be defined for each type, which may germinate and establish as seedlings already in the first time step.

C.2 Germination of seeds

Before seeds can germinate from the seed pool and establish successfully, light and space conditions are checked. Per type a minimum number of seeds can be withheld in the seed pool, which is by default set to 0.

For determining the light conditions, the incoming irradiance on the floor is divided by the incoming irradiance above canopy (see section E for their calculation). This results in the percentage of incoming irradiance on the floor I_{floor}, which is possibly reduced due to shading of already existing individuals. Dependent on a minimum percentage of light I_{seed} required for seed germination and seedling establishment for each type, it is checked

whether I_{floor} is sufficient:

$$N_{germ} = \begin{cases} N_{pool} & ,I_{floor} \geq I_{seed} \\ 0 & ,I_{floor} < I_{seed} \end{cases}, \tag{9}$$

whereby N_{germ} is the number of germinated seedlings.

If light requirements are not sufficient for seeds of a specific type, they remain in the seed pool and may germinate in future time step as far as conditions become favorable. By this, seeds may accumulate in the seed pool if light conditions remain unfavorable over a period of time.

Seeds waiting in the seed pool for favorable germination conditions may be affected by seed pool mortality. For each type a mortality rate M_{pool} [$^1/_{yr}$] is defined prior to the start of the simulation. A rate of $M_{pool} = 0$ represents, for example, an unlimited accumulation of seeds in times of unfavorable conditions. In contrast, a rate of $M_{pool} = 1$ would not allow any accumulation of seeds in the seed pool.

The density of germinated seedlings can be additionally regulated. Thereby, for each type and patch the number N_{germ} is truncated at a predefined value max_{dens}.

C.3 Establishment of seedlings

If light requirements are fulfilled for successful seedling germination, it is secondly checked whether enough space is available for their establishment. Germinated seedlings start with a predetermined stem diameter D_{min}, irrespective of type or species. Using the chosen functional relationships describing the geometry of an individual (see section B), their corresponding height H_{min} can be calculated. If space at the respective height is already filled by more than 100% with existing individuals, none of the germinated seedlings would be able to establish:

$$N_{est} = \begin{cases} N_{germ} & ,CCA_l < 1 \\ 0 & ,CCA_l \geq 1 \end{cases}, \tag{10}$$

whereby N_{est} is the number of successfully established seedlings and CCA_l denotes the cumulative crown area at the height layer l (of width Δh [m]) which correspond to H_{min}:

$$l = \left\lfloor \frac{H_{min}}{\Delta h} \right\rfloor. \tag{11}$$

See section D for the calculation of the cumulative crown area CCA of all height layer of the aboveground discretized space.

Table 3: *Summary of the type-specific recruitment parameters for all plant functional types (PFT).*
References: [1] *Rüger, 2006* [2] *calibrated*

Symbol	PFT 1	PFT 2	PFT 3	PFT 4	PFT 5	PFT 6	
I_{seed}	0.03	0.01	0.05	0.20	0.03	0.20	[1]
N_{seed}	30	156	21	300	2	200	[2]
D_{min}			0.02				[2]

D Mortality

In FORMIND 3.0 trees can die due to different reasons. The following different types of mortality occur in a serial way:

- crowding mortality caused by limited space
- background mortality M_B
- mortality caused by disturbances due to a falling tree

Individual trees of the same type and age, which are located in the same patch, are summarized in this section by a so-called **cohort**. Each cohort is uniquely described by its type, the number of identical trees (N), their age and the size of one single tree (i.e. aboveground biomass). In this section, the number of identical trees in a cohort change due to mortality processes. In the following, we describe these types of mortality in more detail.

D.1 Crowding mortality

Crowding occurs, if at any height layer the cumulative crown area of all trees on a patch exceeds A_{patch}. At first, the cumulative crown area CCA [m^2/m^2] of all trees on a patch is calculated for each height layer i relative to the patch area A_{patch}:

$$CCA_i = \frac{1}{A_{patch}} \cdot \sum_{\substack{all\ individuals \\ with\ l_{min} \leq i \leq l_{max}}} C_A, \qquad (12)$$

where C_A is the crown area of a tree (see section B). Thereby, each tree occupies only a limited amount of height layers (i.e. between layer l_{min} and l_{max}) defined by the individual's crown length C_L [m] and its height H [m]:

$$l_{max} = \left\lfloor \frac{H}{\Delta h} \right\rfloor \qquad (13)$$

$$l_{min} = \left\lfloor \frac{H - C_L}{\Delta h} \right\rfloor \qquad (14)$$

Mortality due to crowding is calculated per tree represented by a reduction factor R_c [-]. This individual reduction factor is calculated based on those height layers, which the individual's crown is occupying (Fig. 4).

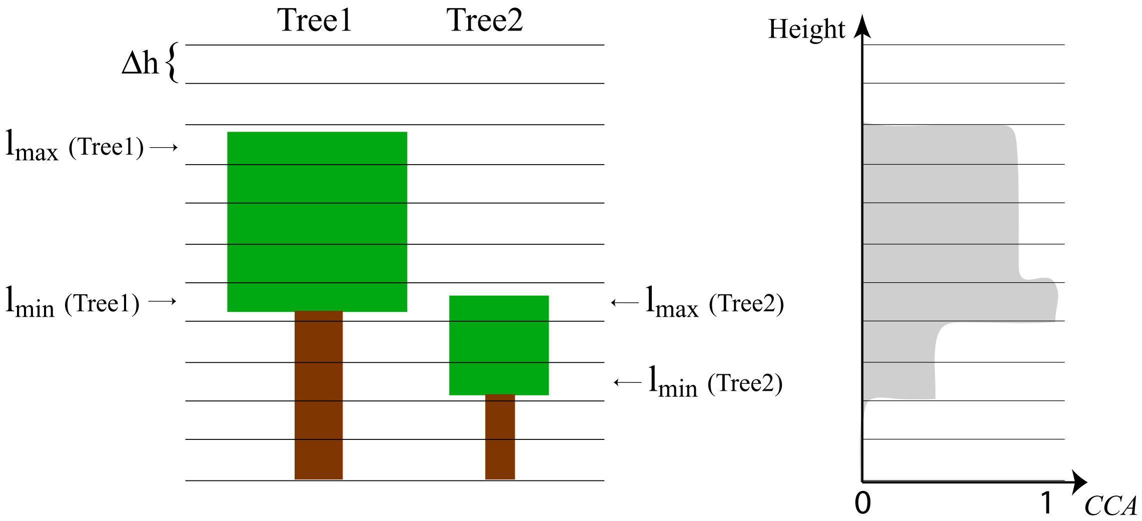

Figure 4: *Illustration of crowding on the example of two single trees . The limits of each crown are shown by l_{min}(Tree1), l_{max}(Tree1), l_{min}(Tree2) and l_{max}(Tree2). The vertically discretized aboveground space into height layers of width Δh [m] is coloured differently according to the sum of the crown projection areas of both individuals occupying the layers. The darker the colour is, the more crowns occupy the respective height layer. This is calculated by the cumulative crown area CCA [-] relative to the patch area, which is illustrated on the right side. The maximum of CCA is used to calculate the reduction factor R_c for each individual. In this example, the reduction factor for each of both trees is calculated based on the 5. height layer from the bottom (equal to layer l_{min}(Tree1) and l_{max}(Tree2)).*

The reduction factor R_c is determined by the reciprocal of the maximum cumulative crown area according to those height layers between the individual limits l_{min} and l_{max}:

$$R_c = \frac{1}{\max\limits_{i \in [l_{min};l_{max}]} (CCA_i)}. \tag{15}$$

If the maximum cumulative crown area of any height layer, which the individual's crown is occupying, exceeds A_{patch} (i.e. $CCA_i > 1$), the individual reduction factor R_c falls below the threshold of 0.99. In this case, the number of dying identical trees per cohort N_C is calculated by:

$$N_C = N\ (1 - R_c). \tag{16}$$

Mortality due to crowding (or self-thinning) can be interpreted as competition for space. For the purpose of time saving, the reduction factor R_c is calculated not directly before crowding mortality occurs. The vertical discretization of the aboveground space is not only important for the calculation of the reduction factor R_c of individuals, but also for the light climate calculations. For this purpose, we move the calculation of R_c to that of the light climate (see chapter E).

D.2 Mortality occurring in each time step

In contrast to the event-driven crowding mortality, a mortality rate per tree is activated in each time step t_y. This mortality rate M is calculated as the sum of the background mortality rate M_B and two further mortality rates dependent on the stem diameter M_D as well as its increment M_I:

$$M = M_B + M_D + M_I. \tag{17}$$

The background mortality M_B [1/yr] is a type-specific constant input parameter.

The mortality rate M_D dependent on the stem diameter is inactive in this model version:

$$M_D = 0. \tag{18}$$

The mortality rate M_I dependent on the increment of the stem diameter per time step is inactive in this model version:

$$M_I = 0. \tag{19}$$

The trees per patch die according to their mortality rate M - either stochastically or deterministically.

Deterministic dying is active if the number of individuals per cohort is greater than a predefined number N_M **and** if the stem diameter of each individual is smaller than a predefined threshold D_M. In this case, the number of dying trees per cohort is determined by:

$$N_Y = N \cdot M, \tag{20}$$

where N is the number of trees per cohort, N_Y is the number of dying trees per cohort and M is the calculated mortality rate per time step t_y. The number of dying trees N_Y is rounded by $\lfloor N_Y + 0.5 \rfloor$.

In the contrary case, stochastic dying is performed (i.e. $N < N_M$ **or** $D > D_M$). That means, for each tree the mortality rate M represents its probability of dying (i.e. by comparing a random number from a uniform distribution in the range of [0;1] with the mortality rate M):

$$N_Y = \sum_{j=1}^{N} \delta_{rM}, \tag{21}$$

where N is the number of trees per cohort, N_Y is the number of dying trees per cohort, M is the calculated mortality rate per time step t_y and r is a random number from a uniform distribution in the range of [0;1]. The symbol δ_{rM} is defined as:

$$\delta_{rM} = \begin{cases} 1 & ,r \leq M \\ 0 & ,r > M \end{cases} \tag{22}$$

Tree falling and resulting damages of affected trees

If one tree falls, neighboring trees can be destroyed as well. A dying tree falls down with probability f_{fall}. The falling target depends on falling direction and on tree height H. Falling direction DIR (drawn from a uniform distribution in the range of [0°,360°]) is chosen randomly. The target coordinates of the falling tree (x_{fall}, y_{fall}) are determined in the following way:

$$x_{fall} = x_{tree} + H\ sin\left(2\ \pi\ \frac{DIR}{360}\right) \tag{23}$$

$$y_{fall} = y_{tree} + H\ cos\left(2\ \pi\ \frac{DIR}{360}\right) \tag{24}$$

whereby (x_{tree}, y_{tree}) is the standing position of the falling tree . With this target coordinates the affected patch is determined. All smaller trees (tree height $< H$) in this target patch are dying with a probability M_{dam}:

$$M_{dam} = C_A / A_{patch}, \tag{25}$$

whereby C_A is the crown area of the falling tree and A_{patch} the area of the target patch.

The trees in the target patch die according to the damage rate M_{dam} - either stochastically or deterministically. Deterministic dying is active if the number of trees per cohort is greater than 100. In this case, the number of dying trees per cohort N_F is determined by multiplying number of trees N per cohort with damage rate M_{dam}.

$$N_F = N \cdot M_{dam}, \tag{26}$$

The number of dying trees N_F is rounded by $\lfloor N_F + 0.5 \rfloor$.

In the contrary case (less than 100 trees per cohort), stochastic dying is performed. That means, for each tree the damage rate M_{dam} represents its probability of dying (i.e. by comparing a random number from a uniform distribution in the range of $[0;1]$ with the damage rate).

$$N_F = \sum_{j=1}^{N} \delta_{rM_{dam}}, \tag{27}$$

where N is the number of trees per cohort, N_F is the number of dying trees per cohort, M_{dam} is the damage rate per time step t_y and r is a random number from a uniform distribution in the range of $[0;1]$. The symbol $\delta_{rM_{dam}}$ is defined as:

$$\delta_{rM_{dam}} = \begin{cases} 1 & ,r \leq M_{dam} \\ 0 & ,r > M_{dam} \end{cases} \tag{28}$$

Table 4: *Summary of the type-specific mortality parameters for all plant functional types (PFT). References:* [1] *calibrated* [2] *Brokaw, 1985*

Symbol	PFT 1	PFT 2	PFT 3	PFT 4	PFT 5	PFT 6	
M_B	0.015	0.030	0.029	0.040	0.021	0.045	[1]
f_{fall}			0.40				[2]

D.3 Overall change in number of trees per cohort

Overall, per time step t_y and for each cohort the change in the number of trees per cohort N is determined by:

$$dN/dt = -(N_C + N_Y + N_F), \tag{29}$$

where N_C is the number of trees dying due to crowding, N_Y is the number of trees dying due to regularly mortality and N_F is the number of trees dying due to damages caused by a falling tree .

The amount of above ground carbon S_{mort} [$^{t_C}/_{\mathrm{ha}}$], which results from the death of trees within the current time step is calculated by:

$$S_{mort} = 0.44 \cdot \sum_{all\ cohorts} (N_C + N_Y + N_F) \cdot B, \tag{30}$$

where B is the above ground biomass of the tree (see section B). We assume that 1 g organic dry matter contains 44 % carbon [Larcher, 2001].

E Competition and environmental limitations

E.1 Light climate

A single tree on a patch receives full incoming radiation. An increasing number of individual trees of differing heights on a patch results in shading within the canopy. Higher trees partly intercept radiation, which is not available for smaller individuals. Thus, with decreasing height from the canopy down to the ground, radiation is increasingly attenuated. We call this vertical distribution of light availability within a patch 'light climate'.

To calculate the light availability in different heights within the canopy, the vertical discretization of the above-ground space is used (i.e. height layers of constant width Δh). For each patch and height layer, the leaf area accumulated by all trees on the patch is calculated. Each tree contributes parts of its crown leaf area to those height layers, which are occupied by its crown (i.e. height layers from l_{min} to l_{max}). These limits are determined by the individual's crown length C_L and its height H:

$$l_{max} = \left\lfloor \frac{H}{\Delta h} \right\rfloor \tag{31}$$

$$l_{min} = \left\lfloor \frac{H - C_L}{\Delta h} \right\rfloor . \tag{32}$$

The number of height layers a tree is occupying by its crown ($\#_{layer}$) can then be calculated by:

$$\#_{layer} = l_{max} - l_{min}. \tag{33}$$

For those height layers between l_{min} and l_{max}, an individual's leaf area contributes equally to each layer i:

$$\bar{L}_i = \frac{LAI \cdot C_A}{\#_{layer}}, \tag{34}$$

whereby $\bar{L}_i$ [m^2] represents the contribution of an tree 's leaf area to the layer i, LAI [-] is the leaf area index of the tree (see section B.6) and C_A [m^2] is crown projection area of the tree 's crown. The multiplication of LAI by C_A results in the leaf area in [m^2] of an single tree .
Summing up all contributions of the trees ' leaf area per patch to their respective occupied height layers and relative to the patch area, results in the patch-based leaf area index $\hat{L}_i$ [-] per layer i:

$$\hat{L}_i = \frac{1}{A_{patch}} \sum_{\substack{all\ individuals \\ with\ l_{min} \leq i \leq l_{max}}} \bar{L}_i, \tag{35}$$

where $\bar{L}_i$ [m^2] represents the leaf area contribution of an tree to the height layer i and A_{patch} [m^2] denotes the area of a patch.

Using this information, the radiation each tree is able to intercept can be determined. Light attenuation through the canopy is calculated using the approach of [Monsi and Saeki, 1953]. The incoming radiation I_{ind} on top of a tree (i.e. on top of the height layer l_{max} the tree is reaching) is calculated by:

$$I_{ind} = I_0 \cdot exp\left(-k \cdot \sum_{i>l_{max}} \hat{L}_i\right), \qquad (36)$$

where the sum in the exponent accumulates the patch-based leaf area indices of all height layers above the individual's height. The parameter k denotes the light extinction coefficient [-] of a tree , I_0 [μmol (photons)/m^2 s] is the daily radiation above canopy averaged from sunrise to sunset during the vegetation period and $\hat{L}_i$ [-] represents the patch-based leaf area index of height layer i.

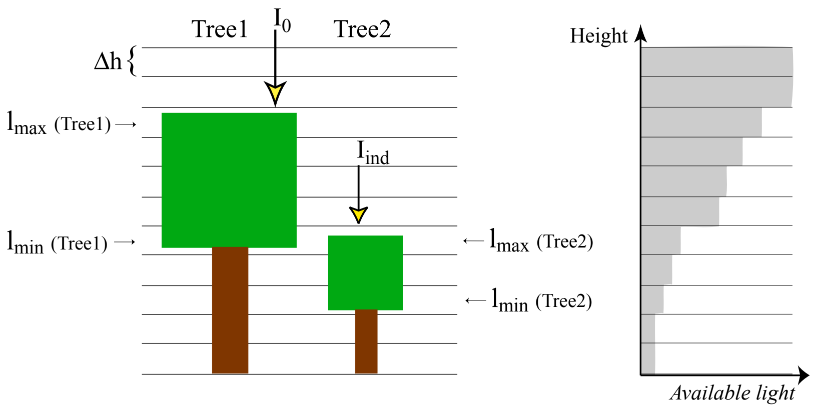

Figure 5: *Illustration of the light climate on the example of two single trees . The limits of each crown are shown by l_{min} (Tree1), l_{max}(Tree1), l_{min}(Tree2) and l_{max}(Tree2). The vertically discretized aboveground space into height layers of width Δh [m] is coloured differently according to the available radiation. The lighter the colour is, the more attenuated the radiation is, which results from the absorption by higher individuals' leaves. On the right hand side the decrease of available light from the canopy to the floor is illustrated by the grey polygon. Thereby, attenuation is greatest in the height layer both trees occupy by their crowns (i.e. layer l_{min}(Tree1) and l_{max}(Tree2)).*

By determining the available radiation for each single tree , competition for light between trees is considered.

Table 5: *Summary of the light climate parameters.*
References: [1]*climate data provided by DFG Kili project (T. Nauss)* [2]*Huth and Ditzer, 2000*

Symbol	value	
I_0	860	[1]
k	0.7	[2]

F Growth of a tree

F.1 Interim photosynthesis

Based on the incoming irradiance on top of a tree I_{ind} (see section E), organic dry matter is produced via gross photosynthesis. In this section the interim photosynthesis is calculated without reduction due to limited soil water availability nor temperature effects.

The interim gross photosynthesis P_{ind} of an individual is modelled using the approach of [Thornley and Johnson, 1990]. It is based on the single-leaf photosynthesis modelled by a Michaelis-Menten function – a typical saturation function describing the relation between the radiation I_{leaf} available on top of a leaf and its gross photosynthetic rate P_{leaf}:

$$P_{leaf}(I_{leaf}) = \frac{\alpha \cdot I_{leaf} \cdot p_{max}}{\alpha \cdot I_{leaf} + p_{max}}, \tag{37}$$

where α is the quantum efficiency, also known as the initial slope of the type-specific light response curve, I_{leaf} is the incoming irradiance on top of the surface of a single leaf within the individual's crown and p_{max} is the maximum leaf gross photosynthetic rate.

To obtain the incoming irradiance on top of the surface of a single leaf I_{leaf}, the available irradiance I_{ind} on top of the entire individual has to be modified:

$$I_{leaf}(L) = \frac{k}{1-m}\, I_{ind} \cdot e^{-k \cdot L}, \tag{38}$$

where k [-] is the type-specific light extinction coefficient, m [-] represents the transmission coefficient and I_{ind} denotes the available incoming irradiance on top of a tree .

The first part $\frac{k}{1-m}\, I_{ind}$ in eqn. (38) is correcting the incoming irradiance in order to obtain those parts, which can be absorbed by a leaf. The second part $e^{-k \cdot L}$ in eqn. (38) accounts for self-shading within the individual's crown. As the leaves of an individual are assumed to be homogeneously distributed within its crown, some leaves will be shaded by higher ones within the crown. Thereby, $L = 0$ represents the top of the individual and $L = LAI$ represents the bottom of the individual's crown with LAI being its leaf area index (see section B).

To obtain the interim gross photosynthetic rate of a tree per year P_{ind}, the single-leaf photosynthesis of eqn. (37) is integrated over the individual's leaf area index LAI (see section B):

$$P_{ind} = \int_0^{LAI} P_{leaf}(I_{leaf}(L))dL. \tag{39}$$

The integration results in the interim photosynthesis of an tree per year [Thornley and Johnson, 1990]:

$$P_{ind} = \frac{p_{max}}{k} \cdot ln \frac{\alpha\ k\ I_{ind} + p_{max}(1-m)}{\alpha\ k\ I_{ind}\ e^{-k \cdot LAI} + p_{max}(1-m)}. \tag{40}$$

To convert the interim photosynthesis P_{ind} from $[\mu mol_{CO_2}/m^2 s]$ to $[t_{ODM}/y]$, P_{ind} has to be multiplied by the individual's crown area C_A (see section B), the type-specific photosynthetic active period φ_{act} and finally a conversion factor c_{odm}:

$$P_{ind} \cdot C_A \cdot 60 \cdot 60 \cdot l_{day} \cdot \varphi_{act} \cdot \varphi_{odm}, \tag{41}$$

where the multiplication by $60 \cdot 60$ accounts for the conversion from seconds to hours. The factor l_{day} [h] represents the mean day length during the vegetation period φ_{act} [d] (see section E). The conversion factor $\varphi_{odm} = 0.63 \cdot 44 \cdot 10^{-12}$ includes the molar mass of CO_2, the conversion from g to t and the conversion from CO_2 to organic dry mass ODM [Larcher, 2001].

F.2 Gross primary production

The gross primary production GPP of a tree is calculated from the interim photosynthesis P_{ind} $[t_{ODM}/y]$ (see section F.1):

$$GPP = P_{ind}\ \varphi_T\ \varphi_W, \tag{42}$$

where φ_W denotes the reduction factor accounting for limited soil water and φ_T represents the limitation factor of air temperature effect. Both factors range between 0 and 1 and thus, only reducing GPP in times of unfavorable conditions. Climatic effects on GPP are not active in this model version (both factors are 1).

Table 6: *Summary of the type-specific photosynthetic parameters for all plant functional types (PFT).*
References: [1] climate data provided by DFG Kili project (T. Nauss) [2] Cai et al., 2005 [3] Fischer et al., 2014 [4] Dislich et al., 2009 [5] Zhang et al., 2012 [6] Rüger, 2006 [7] calibrated

Symbol	PFT 1	PFT 2	PFT 3	PFT 4	PFT 5	PFT 6	
l_{day}			12				[1]
φ_{act}			360				[1]
p_{max}	2.0	3.1	6.8	11.0	7.0	12.0	[2,3,4,5]
α	0.36	0.28	0.23	0.20	0.30	0.20	[2,5,6,7]

F.3 Biomass increment of a tree

Gross primary production GPP of eqn. (42) is first used for the maintenance of the already existing aboveground biomass of an tree . Costs for maintenance are modelled as biomass losses in terms of maintenance respiration R_m $[t_{ODM}/y]$. The remaining productivity $(GPP - R_m)$ is then available for growth of new aboveground biomass. Costs for the production of new structural tissue are modelled also as biomass losses in terms of growth respiration. This results in the net productivity ΔB [Dislich et al., 2009]:

$$\Delta B = (1 - r_g)\ (GPP - R_m), \tag{43}$$

where r_g [-] represents a constant parameter describing the fraction of $(GPP - R_m)$ attributed to growth respiration. In contrast, maintenance respiration R_m is modelled proportionally to the already existing aboveground biomass of a tree (see section F.4).

F.4 Maintenance respiration

The maintenance respiration R_m of a tree is calculated inversely by rearranging eqn. (43):

$$R_m = GPP - \frac{\Delta B}{1 - r_g}. \tag{44}$$

Maintenance respiration R_m is further modelled proportional to the already existing aboveground biomass B $[t_{ODM}]$ of an individual:

$$R_m = \kappa_T \cdot r_m \cdot B, \tag{45}$$

where r_m denotes the maintenance respiration rate [$^1/_y$] and κ_T represents a limitation factor dependent on air temperature. Climatic effects on respiration are not active in this model version ($\kappa_T = 1$).

Combining equation (44) with equation (45) and arranging in terms of the respiration rate r_m results in:

$$r_m = \frac{1}{B \cdot \kappa_T} \cdot \left(GPP - \frac{\Delta B}{1 - R_g} \right). \tag{46}$$

The maintenance respiration rate r_m of eqn. (46) is calculated using the assumption of full resource availability. Thereby, it is assumed that full resource availability (i.e. no limitation by shading, soil water or air temperature) results in the observed maxima of field measurements of stem diameter increments:

$$r_m = \frac{1}{B} \cdot \left(P_{ind}(I_0) - \frac{B(D + g(D)) - B}{(1 - R_g)} \right), \tag{47}$$

where this equation can be obtained by substituting in eqn. (46) (i) κ_T by 1, (ii) GPP by the gross productivity under full resource availability $P_{ind}(I_0)$ (see eqn. 41) with I_0 as the full available incoming irradiance and (iii) ΔB by the biomass increment derived from the maximum stem diameter increment under full resource availability $D + g(D)$ using the individual's geometry (see section B). See section F.5 for different modelling approaches of the *maximum diameter growth curve* $g(D)$.

F.5 Maximum diameter growth curve

In the field, diameter increments can be determined by calculating the differences between two measurements of the stem diameter per tree (at two distinct observation dates). The increments are then usually plotted with the measured stem diameter of the first observation date to get an impression of how much a tree of stem diameter D is able to increase (see Fig. 6 for an example).

Such point clouds as illustrated in Fig. 6 can be described by functional relationships. Please note, that you have to adjust the increments according to a time step of 1 year. That means, if there is a period of e.g. 5 years between both observation dates of stem diameter measurements, you would have to correct the increments with respect to the smaller time scale.

Only a few information of the measured diameter increment curve are needed to derive:

- maximum diameter increment ΔD_{max} [m/y]
- stem diameter $D_{\Delta D_{max}}$ [% of D_{max}], which reaches ΔD_{max}
- maximum diameter increment $\Delta D_{D_{min}}$ [% of ΔD_{max}] of the smallest possible tree (with $D = D_{min}$)
- maximum diameter increment $\Delta D_{D_{max}}$ [% of ΔD_{max}] of the biggest possible tree (with $D = D_{max}$)

Based on these characteristics, the coefficients of the growth function $g(D)$ can be calculated explicitly. In this model version, a Chanter approach is chosen as maximum growth curve.

Chanter approach

This approach describes the growth function $g(D)$ as follows:

$$g(D) = a_0 \cdot D \cdot \left(1 - \frac{D}{D_{max}}\right) \cdot e^{-a_1 \cdot D}, \tag{48}$$

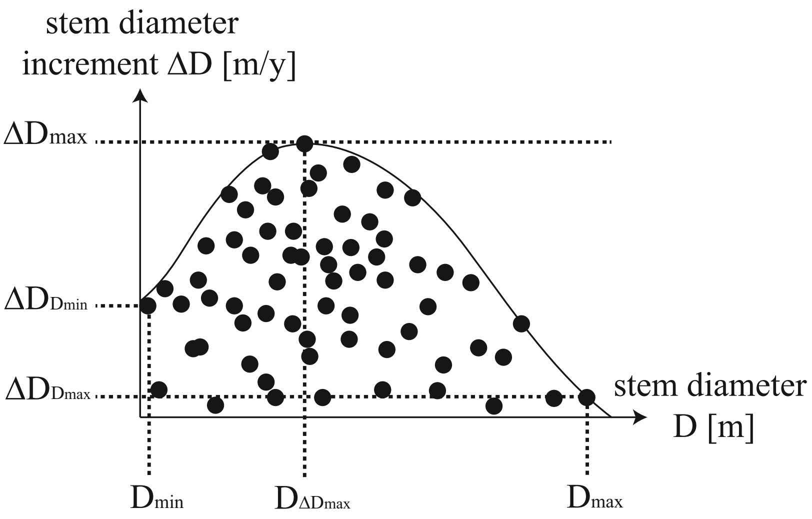

Figure 6: *Illustration of a measured diameter growth curve. Points represent illustrative measurements. The solid line represents a fitted growth function to the maximum values of the measurements. Dotted lines show important characteristics which would be needed for the first approach.*

where a_0 and a_1 are the type-specific coefficients, which are calculated by:

$$a_0 = \frac{e^{\frac{D_{max}-2\cdot(D_{\Delta D_{max}}\cdot D_{max})}{D_{max}-(D_{\Delta D_{max}}\cdot D_{max})}} \cdot D_{max} \cdot \Delta D_{max}}{(D_{max}-(D_{\Delta D_{max}}\cdot D_{max}))\cdot(D_{\Delta D_{max}}\cdot D_{max})}$$

$$a_1 = \frac{D_{max}-2\cdot(D_{\Delta D_{max}}\cdot D_{max})}{D_{max}\cdot(D_{\Delta D_{max}}\cdot D_{max})-(D_{\Delta D_{max}}\cdot D_{max})^2},$$

whereby D_{max} is calculated out of maximum height (see section B.7).
Please note, when determining the type-specific coefficients prior to the start of the simulation, that the curve represents growth under full resource availability. That means, not all measurements should be fitted, but only the maximum diameter increments (see Fischer, 2010 p. 55 for an example).

Table 7: *Summary of the type-specific growth and respiration parameters for all plant functional types (PFT).*
References: [1]*calibrated* [2]*Ryan, 1991*

Symbol	PFT 1	PFT 2	PFT 3	PFT 4	PFT 5	PFT 6	
ΔD_{max}	0.012	0.012	0.019	0.029	0.011	0.029	[1]
$D_{\Delta D_{max}}$	33	34	23	60	33	60	[1]
r_g			0.25				[2]

G Carbon cycle

The calculation of the carbon cycle in FORMIND 3.0 uses a simple compartment approach consisting of the following explicit carbon stocks:

- living forest stock, which equals the amount of carbon of alive trees
- deadwood stock S_{dead}, which equals the amount of carbon of dead trees
- slow decomposing soil stock S_{slow}, which accounts for the amount of carbon decomposed slowly from the deadwood stock
- fast decomposing soil stock S_{fast}, which accounts for the amount of carbon decomposed fast from the deadwood stock

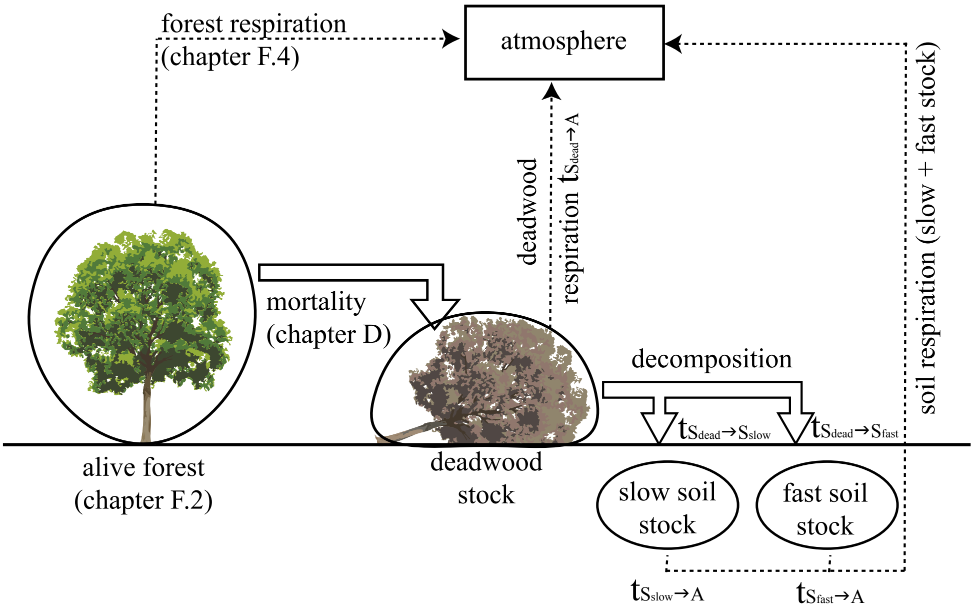

Figure 7: *Schematic visualization of the carbon cycle in FORMIND 3.0 . Circles represent explicit carbon stocks and the rectangle indicates the atmosphere. Dotted arrows show carbon released to the atmosphere from the respective stock and block arrows show carbon transitions between the respective explicit carbon stocks.*

The dynamics of the living forest stock (i.e. carbon storage in form of growth and carbon releases as respiration) are described earlier in section F. The dynamics of the remaining stocks is described by a set of differential equations:

$$\begin{aligned}
\frac{\mathsf{d}S_{dead}}{\mathsf{d}t} &= S_{mort} - (t_{S_{dead}\to A} + t_{S_{dead}\to S_{slow}} + t_{S_{dead}\to S_{fast}}) \cdot S_{dead} \\
\frac{\mathsf{d}S_{slow}}{\mathsf{d}t} &= t_{S_{dead}\to S_{slow}} \cdot S_{dead} - t_{S_{slow}\to A} \cdot S_{slow} \\
\frac{\mathsf{d}S_{fast}}{\mathsf{d}t} &= t_{S_{dead}\to S_{fast}} \cdot S_{dead} - t_{S_{fast}\to A} \cdot S_{fast}
\end{aligned}$$

where the parameters $t_{S_{dead}\to A}$, $t_{S_{slow}\to A}$ and $t_{S_{fast}\to A}$ denote transition rates in [$^1/_{\text{yr}}$] of released carbon from the respective soil stocks to the atmosphere. The parameter $t_{S_{dead}\to S_{slow}}$ and $t_{S_{dead}\to S_{fast}}$ represent in turn decomposition rates of deadwood material in [$^1/_{\text{yr}}$]. The variable S_{mort} [$^{tC}/_{\text{ha}}$] represents the carbon of all trees dying within the current time step (see section D).

G.1 Determining the transition rates

The transition rates depend on how fast microorganisms can decompose the fallen litter or dead trees. For describing the decomposition rates, we use an approach presented earlier by Sato et al. [2007]. The annual decomposition rate $t_{S_{dead}\to}$ for the deadwood stock is calculated as follows:

$$t_{S_{dead}\to} = \min\left(1.0, \frac{10^{-1.4553+0.0014175\cdot AET}}{12}\right), \tag{49}$$

where AET is considered as the actual evapotranspiration in the previous year in mm. The variable AET is given as a fixed input parameter (see Table 8).

The annual decomposition rate $t_{S_{dead}\to}$ is modelled as the sum of all transitions rates of the deadwood pool S_{dead}:

$$t_{S_{dead}\to} = t_{S_{dead}\to A} + t_{S_{dead}\to S_{slow}} + t_{S_{dead}\to S_{fast}} \tag{50}$$

According to [Sato et al., 2007] 70 % of the carbon of decomposing deadwood biomass (i.e. litter) is directly released to the atmosphere, while the remaining 30 % are transferred to the slow and fast decomposing soil stocks. In detail, 98.5 % of the remaining carbon is transferred to the fast soil stock and 1.5 % to the slow soil stock. We then calculate the specific transition rates as follows:

$$\begin{aligned}
t_{S_{dead}\to A} &= 0.7 \cdot t_{S_{dead}\to} \\
t_{S_{dead}\to S_{slow}} &= 0.015 \cdot 0.3 \cdot t_{S_{dead}\to} \\
t_{S_{dead}\to S_{fast}} &= 0.985 \cdot 0.3 \cdot t_{S_{dead}\to}
\end{aligned}$$

G.2 The Net Ecosystem Exchange (NEE)

The NEE is the carbon net flux of the forest. We define the NEE $[{}^{t_C}/_{\text{ha yr}}]$ as follows:

$$NEE = C_{GPP} - C_R - t_{S_{dead} \to A} \cdot S_{dead} - t_{S_{slow} \to A} \cdot S_{slow} - t_{S_{fast} \to A} \cdot S_{fast}, \tag{51}$$

where S_{dead} $[{}^{t_C}/_{\text{ha}}]$ denotes the deadwood carbon pool, S_{slow} $[{}^{t_C}/_{\text{ha}}]$ and S_{fast} $[{}^{t_C}/_{\text{ha}}]$ the soil carbon stock (i.e. slow and fast decomposing), $t_{x \to A}$ $[{}^{1}/_{\text{yr}}]$ the corresponding transition rates of released carbon from the respective stock x resulting from the microbiological respiration (cf. section G) and C_{GPP} $[{}^{t_C}/_{\text{ha yr}}]$ is the carbon captured in the gross primary productivity of the living forest (cf. section F.2), C_R $[{}^{t_C}/_{\text{ha yr}}]$ is the carbon released by the total respiration of the living forest (i.e. for maintenance and growth). We also assume here that 1 g organic dry matter contains 44 % carbon, which results in:

$$\begin{aligned} C_{GPP} &= 0.44 \cdot \sum_{all\ trees} GPP \\ C_R &= 0.44 \cdot \sum_{all\ trees} (R_m + R_g \cdot (GPP - R_m)). \end{aligned}$$

If the NEE is positive (i.e. $NEE > 0$), the forest is considered to be a carbon sink. If the NEE is negative (i.e. $NEE < 0$), the forest is considered to be a carbon source.

Table 8: *Summary of the parameters concerning the carbon cycle.*
References: [1] *climate data provided by DFG Kili project* [2] *Sato et al., 2007*

Symbol	Value	
AET	1,300	1
$t_{S_{slow} \to A}$	$^1/_{750}$	2
$t_{S_{fast} \to A}$	$^1/_{15}$	2

H Input parameter and variables

Table 9: *General input parameter of the simulation.*

Symbol	Description	Unit
A_{area}	Simulation area	ha
A_{patch}	Patch area	m^2
$\#_{patches}$	Number of patches per simulation area	
t_y	time step	yr

Table 10: *Geometrical input parameter.*

Symbol	Description	Unit
h_0, h_1, h_2	Height-Stem diameter-Relationship	-
c_{l0}, c_{l1}, c_{l2}	Crown length-Height-Relationship	-
c_{d0}, c_{d1}, c_{d2}, c_{d3}	Crown diameter-Stem diameter-Relationship	-
ρ	Wood density	t_{ODM}/m^3
σ	Ratio of total aboveground biomass to stem biomass	-
f	form factor	-
f_0, f_1, f_2	Form factor-Stem diameter-Relationship	-
b_0, b_1, b_2	Biomass-Stem diameter-Relationship	-
l_0, l_1	LAI-Stem diameter-Relationship	-
D_{max}	Maximum stem diameter	m
H_{max}	Maximum height	m
B_{max}	Maximum biomass	t_{ODM}

Table 11: *Recruitment and establishment input parameter.*

Symbol	Description	Unit
N_{seed}	Global in-growth rate	$1/\text{ha yr}$
N_{init}	Initial seed number in seed pool	$1/\text{patch}$
D_{rep}	Minimum stem diameter of a recruiting mother tree	m
f_{disp}	Dispersal kernel	-
$dist$	Average dispersal distance	m
σ	Ratio of total aboveground biomass to stem biomass	-
I_{seed}	Percentage of incoming radiation at floor required for germination	%
M_{pool}	Seed pool mortality rate	$1/\text{yr}$
max_{dens}	Maximum number of germinated seedlings	$1/\text{patch}$
D_{min}	Stem diameter of a germinated seedling	m

Table 12: *Mortality input parameter.*

Symbol	Description	Unit
M_B	Basic mortality rate	$1/\text{yr}$
m_{d0}, m_{d1}	Mortality rate dependent on stem diameter	-
m_{i0}, m_{i1}, m_{i2}	Mortality rate dependent on stem diameter increment	-
N_M	Min. number of individuals at which stochastic dying is performed	$1/\text{cohort}$
D_M	Max. stem diameter below which stochastic dying is performed	m
t_{meadow}	Time ...	yr
t_{regrow}	Time ...	yr

Table 13: *Light climate and photosynthesis input parameter and variables.*

Symbol	Description	Unit
Δh	Width of layers of aboveground vertical space discretization	m
$\#_{layer}$	Number of layer of aboveground vertical space discretization	
I_0	Incoming irradiance on top of canopy	$\mu mol_{photon}/m^2\ s$
k	Light extinction coefficient	-
α	Initial slope of light response curve	$\mu mol_{CO_2}/\mu mol_{photon}$
p_{max}	Maximum leaf gross photosynthetic rate	$\mu mol_{CO_2}/m^2 s$
m	Transmission coefficient	-
l_{day}	Day length	h
φ_{ODM}	Conversion factor	$t_{ODM}/\mu mol_{CO_2}$

Table 14: *Water module input parameter and variables.*

Symbol	Description	Unit
PR	Precipitation	mm/h
K_L	Interception constant	mm/h
POR	Soil porosity	mm/h
K_s	Fully saturated conductivity	mm/h
Θ_{res}	Residual soil water content	mm/h
λ	Pore size distribution index	-
WUE	Water-use-efficiency	t_{ODM}/kg_{H_2O}
PET	Potential evapotranspiration	mm/h
Θ_{soil}^{init}	Initial soil water content at start of simulation	V%
Θ_{pwp}	Permanent wilting point	V%
Θ_{fc}	Field capacity	V%
Θ_{msw}	Minimum soil water content	V%

Table 15: *Temperature input parameter and variables.*

Symbol	Description	Unit
T	Air temperature	$°C$
n	Number of days per time step t_y	$1/t_y$
T_{crit}	Critical temperature for bud-burst	$°C$
k_0, k_1, k_2	Parameter of inhibition factors	-
$T_{CO_2,l}$, $T_{CO_2,h}$	temperature limits of CO_2 assimilation	$°C$
T_{hot}, T_{cold}	monthly mean temperature of warmest and coldest month an individual can cope with	$°C$
T_{ref}	Reference temperature	$°C$
Q_{10}	Base of Q10 function	-

Table 16: *Respiration input parameter and variables.*

Symbol	Description	Unit
R_g	Growth respiration factor	-
$g(D)$	Maximum stem diameter increment (growth) function	-
a_0, a_1, a_2, a_3	Coefficients of the growth function $g(D)$	-
x_i, $i = 1, ..., 8$	Auxillary variables	-
ΔD_{max}	Maximum measured stem diameter increment	m/y
$D_{\Delta D_{max}}$	Stem diameter at which maximum increment is measured	% of D_{max}
$\Delta D_{D_{min}}$	Max. measured stem diameter increment for diameter D_{min}	% of ΔD_{max}
$\Delta D_{D_{max}}$	Max. measured stem diameter increment for diameter D_{max}	% of ΔD_{max}
$\check{I_{ind}}$	Reference irradiance of parameterization climate	$\mu mol_{photon}/m^2 s$
$\check{\varphi_{act}}$	Reference vegetation period of parameterization climate	d
$\check{\varphi_T}$	Reference temperature limitation factor of photosynthesis of parameterization climate	-

I State variables

Table 17: *Geometrical state variables.*

Symbol	Description	Unit
D	Stem diameter at breast height	m
H	Height	m
C_D	Crown diameter	m
C_L	Crown length	m
C_A	Crown projection area	m^2
B	Aboveground biomass	t_{ODM}
LAI	Leaf area index	-
ΔB	Biomass increment per time step	t_{ODM}
ΔD	Diameter increment per time step	m

Table 18: *Recruitment and establishment state variables.*

Symbol	Description	Unit
N_{pool}	Seed pool (i.e number of seeds)	1/patch
N_{germ}	Number of successfully germinated seeds	1/patch
N_{est}	Number of successfully established seedlings	1/patch
x_{ind}, y_{ind}	Random position of a mother tree on a patch	-
x_{seed}, y_{seed}	Position of a dispersed seed	-
I_{floor}	Percentage of incoming irradiance at floor	%

Table 19: *Mortality state variables.*

Symbol	Description	Unit
M_D	Mortality rate dependent on stem diameter	$1/\text{yr}$
M_I	Mortality rate dependent on stem diameter increment	$1/\text{yr}$
M	Mortality rate affecting individuals each time step	$1/\text{yr}$
m_{frag}	Factor changing the mortality rate M due to fragmentation	-
CCA_i, $i = 1, ..., \#_{layer}$	Cumulative crown area per height layer	-
l_{min}, l_{max}	Lower and upper height layer covered by the crown of a single individual	-
R_c	Individual crowding reduction factor	-
N_C	Number of individuals dying due to crowding	$1/\text{cohort}$
N_Y	Number of individuals dying due to mortality per time step	$1/\text{cohort}$
N	Number of alive individuals	$1/\text{cohort}$
δ_{rM}	Auxillary variable	-
N_F	Number of individuals affected by a falling tree	-

Table 20: *Light climate and growth state variables.*

Symbol	Description	Unit
$\overline{L_i}$	Individual leaf area contribution to height layer i	m^2
$\widehat{L_i}$	Patch-based leaf area index	-
I_{ind}	Incoming irradiance on top of an individual	$\mu mol_{photon}/m^2$ s
I_{leaf}	Incoming irradiance on top of the leaf surface (absorbable radiation)	$\mu mol_{photon}/m^2$ s
P_{ind}	Gross photosynthetic rate of an individual	μmol_{CO_2}/yr
P_{leaf}	Gross photosynthetic rate of a single leaf	$\mu mol_{CO_2}/m^2$ s
GPP	Gross productivity of an individual (possibly reduced)	t_{ODM}/yr
R_m	Maintenance respiration	t_{ODM}/yr
r_m	Maintenance respiration rate	1/yr

Table 21: *Water module state variables.*

Symbol	Description	Unit
Θ_{soil}	Soil water content	mm/h
IN	Interception	mm/h
RO	Run-off	mm/h
$RO_{\rightarrow}$	Surface run-off	mm/h
$RO_{\downarrow}$	Sub-surface run-off	mm/h
TR	Transpiration	mm/h
φ_W	Reduction factor of GPP due to limited soil water	-

Table 22: *Temperature state variables.*

Symbol	Description	Unit
φ_{act}	Length of vegetation period	d
φ_T	Limitation factor of GPP by temperature	-
$\varphi_{T,l}$, $\varphi_{T,h}$	Inhibition factors for low and high temperatures	-
κ_T	Factor affecting maintenance respiration rate r_M by temperature	-

Table 23: *Carbon cycle state variables.*

Symbol	Description	Unit
S_{dead}	Carbon stock of deadwood	$^{t_C}/_{\text{patch}}$
S_{slow}	Carbon amount of slow decomposing soil stock	$^{t_C}/_{\text{patch}}$
S_{fast}	Carbon amount of fast decomposing soil stock	$^{t_C}/_{\text{patch}}$
S_{mort}	Carbon amount of individuals dying within the current time step	$^{t_C}/_{\text{patch}}$
$t_{S_{dead}\rightarrow A}$	Transition rate of carbon from deadwood stock S_{dead} to atmosphere A	$^{t_C}/_{\text{patch}}$
$t_{S_{slow}\rightarrow A}$	Transition rate of carbon from slow decomposing soil stock S_{dead} to atmosphere A	$^{t_C}/_{\text{patch}}$
$t_{S_{fast}\rightarrow A}$	Transition rate of carbon from fast decomposing soil stock S_{dead} to atmosphere A	$^{t_C}/_{\text{patch}}$
$t_{S_{dead}\rightarrow}$	Transition rate of carbon from deadwood stock S_{dead} to soil	$^{t_C}/_{\text{patch}}$
$t_{S_{dead}\rightarrow S_{slow}}$	Transition rate of carbon from deadwood stock S_{dead} to slow decomposing soil stock S_{slow}	$^{t_C}/_{\text{patch}}$
$t_{S_{dead}\rightarrow S_{fast}}$	Transition rate of carbon from deadwood stock S_{dead} to fast decomposing soil stock S_{fast}	$^{t_C}/_{\text{patch}}$
NEE	Net ecosystem exchange	$^{t_C}/_{\text{patch}}$
C_{GPP}	Carbon amount of gross productivity per patch	$^{t_C}/_{\text{patch}}$
C_R	Carbon amount released by total respiration per patch	$^{t_C}/_{\text{patch}}$

Table 24: *Disturbances (fire, landslide) state variables.*

Symbol	Description	Unit
N_D	Number of individuals dying due to disturbances	$^{1}/_{\text{cohort}}$
P_{F_1}, P_{F_2}, P_{F_3}, P_{F_4}	Burning probabilities for the 4 fire tolerance levels	-

J Abbreviations

**Symbol**	**Description**
ODM	Organic dry matter
CO_2	Carbon dioxide
C	Carbon
H_2O	Water
sin	Sinus function
cos	Cosinus function
$\lfloor\rfloor$	Round down
e	Exponential function
ln	Logarithm function
cf.	see
e.g.	exempli gratia (for example)
i.e.	id est (that is)
Fig.	Figure
Tab.	Table

References

Brokaw, N.V.L., 1985. Gap-phase regeneration in a tropical forest. Ecology 66, 682–687.

Cai, Z.Q., Rijkers, T., Bongers, F., 2005. Photosynthetic acclimation to light changes in tropical monsoon forest woody species differing in adult stature. Tree Physiology 25, 1023–1031. http://treephys.oxfordjournals.org/content/25/8/1023.full.pdf+html.

Dislich, C., Günter, S.and Homeier, J., Schröder, B., Huth, A., 2009. Simulating forest dynamics of a tropical montane forest in south ecuador. Erdkunde 63, 347–364.

Fischer, R., 2010. Modellierung des Wachstums von Regenwäldern. Untersuchung der Auswirkungen von Trockenstress und Holznutzung auf den tropischen Regenwald am Beispiel des RNI Betampona (Madagaskar). Master's thesis. HTWK Leipzig.

Fischer, R., Armstrong, A., Shugart, H.H., Huth, A., 2014. Simulating the impacts of reduced rainfall on carbon stocks and net ecosystem exchange in a tropical forest. Environmental Modelling & Software 52, 200 – 206.

Huth, A., Ditzer, T., 2000. Simulation of the growth of a lowland dipterocarp rain forest with formix3. Ecological Modelling 134, 1–25.

Larcher, W., 2001. Ökophysiologie der Pflanzen. Leben, Leistung und Stressbewältigung der Pflanzen in ihrer Umwelt. UTB für Wissenschaft, Verlag Eugen Ulmer Stuttgart.

Monsi, M., Saeki, T., 1953. Uber den lichtfaktor in den pflanzengesellschaften und seine bedeutung fur die stoffproduktion. Jpn. J. Bot 17, 22–52.

Nenninger, A., 2006. Oberirdische Biomasse ausgewählter Baumarten eines tropischen Bergregenwaldes in Südecuador. Master's thesis. Technische Universität München.

Rüger, N., 2006. Dynamics and sustainable use of species-rich moist forests. A process-based modelling approach. Ph.D. thesis. Universität Osnabrück.

Rüger, N., Gutierrez, A.G., Kissling, W.D., Armesto, J.J., Huth, A., 2007. Ecological impacts of different harvesting scenarios for temperate evergreen rain forest in southern chile–a simulation experiment. Forest Ecology and Management 252, 52–66.

Ryan, M.G., 1991. Effects of climate change on plant respiration. Ecological Applications 1, 157–167.

Sato, H., Itoh, A., Kohyama, T., 2007. Seib-dgvm: A new dynamic global vegetation model using a spatially explicit individual-based approach. Ecological Modelling 200, 279–307.

Thornley, J.H.M., Johnson, I.R., 1990. Plant and crop modelling: a mathematical approach to plant and crop physiology. Oxford University Press.

Zhang, Q., Chen, Y., Song, L., Liu, N., Sun, L., Peng, C.L., 2012. Utilization of lightflecks by seedlings of five dominant tree species of different subtropical forest successional stages under low-light growth conditions. Tree Physiology 32, 545–553. http://treephys.oxfordjournals.org/content/32/5/545.full.pdf+html.
