# Supplementary material for: Simulating Carbon Stocks and Fluxes of an African Tropical Montane Forest with an Individual-Based Forest Model
Source: PLoS One. 2015 Apr 27;10(4):e0123300. doi: 10.1371/journal.pone.0123300 (PMC4410999; doi:10.1371/journal.pone.0123300)
Supplement: S2 Text — (DOCX) [file pone.0123300.s003.docx]

**S2 Text. Species grouping into plant functional types by species traits.**

All local tree species were grouped into six plant functional types according to their maximum attainable height and their light requirements (see Table 1 in the main text). We used three levels of shade tolerance: shade-tolerant (climax species), shade-intolerant (pioneer species) and intermediate shade-tolerant. Grouping by maximum height was straightforward using inventory data and data from published sources [1]. Shade tolerance, i.e. light requirement, was defined using plant functional traits measured in situ. As data was not available for all species and to verify our results, expert knowledge was employed to confirm the classification (Andreas Hemp, personal communication).

Shade tolerance is an important factor defining demographic patterns in forest ecosystems. Despite decades of research [e.g.,2], no reliable relationship between plant functional traits and shade tolerance has been found yet [3]. Here, we combined a leaf and a stem trait to predict shade tolerance. Taken together, the traits should be able to identify two life strategies of trees associated with shade tolerance: moderately fast-growing canopy species and slow-growing understorey species. We are aware that neither stem dry matter content (dry weight/fresh weight, StDMC in g/g) nor leaf nitrogen content per leaf dry mass (leaf_N_ in mg/g) are causal in enabling shade tolerance; but their correlation with shade tolerance makes it practical to use them to define shade classes. StDMC is known to be low in light-demanding pioneer species and high in slow-growing understorey species [4]. Shade tolerance can also be found in moderately fast-growing canopy species with comparably low StDMC, too. Leaf_N_ is known to be higher in sun leaves than in shade leaves [5], the latter being found in the canopies of understorey species while the former occurs in canopy species. Multiplying both traits, we accounted for both life strategies. We classified all tree species according to the resulting metric (shade index L) into three different groups of shade tolerance. Other species for which traits were not recorded yet were classified by expert knowledge.

$$L=StDMC\cdot Nm$$

Trees with a shade index L < 6 were classified as shade intolerant, species with 6 ≤ L < 9 as medium shade tolerant and species with L ≤ 9 as shade tolerant. An full overview of the grouping of tree species into plant functional types could be found in Table B.1.

| **Species Name** | **StDMC  [g/g]** | **leaf N [mg/g]** | **Shade Class** | **max Height [m]** | **PFT** |
| --- | --- | --- | --- | --- | --- |
| *Alangium chinense* |  |  | intol | 25 | **4** |
| *Aningeria adolfi-friedericii* |  |  | tol | 50 | **1** |
| *Casearia battiscombei* |  |  | tol | 30 | **1** |
| *Celtis durandii* |  |  | med | 25 | **3** |
| *Cyathea manniana* | 0.18 | 28.74 | intol | 15 | **6** |
| *Entandrophragma excelsum* | 0.44 | 22.84 | tol | 70 | **1** |
| *Ficus sur* | 0.35 | 21.27 | med | 25 | **3** |
| *Garcinia tanzaniensis* |  |  | tol | 40 | **1** |
| *Hallea rubrostipulata* |  |  | med | 33 | **3** |
| *Heinsenia diervilleoides* | 0.48 | 29.43 | tol | 25 | **2** |
| *Leptonychia usambarensis* | 0.54 | 26.68 | tol | 15 | **5** |
| *Macaranga capensis var. kilimandscharica* | 0.45 | 18.63 | med | 30 | **3** |
| *Newtonia buchananii* |  |  | tol | 40 | **1** |
| *Polyscias albersiana* |  |  | intol | 15 | **4** |
| *Strombosia scheffleri* | 0.60 | 29.20 | tol | 35 | **1** |

Table B.1: Grouping of tree species into plant functional types (PFT) depending on size class and shade tolerance. Grouping was based on three height classes (<16m, 16-33m, >33m) and three shade tolerance classes (shade-tolerant (climax species) - tol, shade-intolerant (pioneer species) - intol, and intermediate shade-tolerant - med). The shade tolerance classes were determined by species traits: stem dry matter content (StDMC) and nitrogen content in the leaves (leaf N). The traits were not measured for all species yet.

Reference List

1. Turrill WB, Milne-Redhead E (1952) Flora of tropical East Africa.

2. Woods DB, Turner NC (1971) Stomatal response to changing light by four tree species of varying shade tolerance. New Phytol 70: 77-84.

3. Valladares F, Niinemets U (2008) Shade Tolerance, a Key Plant Feature of Complex Nature and Consequences. Annu Rev Ecol Evol Syst 39: 237-257.

4. Lieberman D, Lieberman M (1987) Forest Tree Growth and Dynamics at La Selva, Costa Rica (1969-1982). J Trop Ecol 3: 347-358.

5. Reich PB, Uhl C, Walters MB, Ellsworth DS (1991) Leaf life-span as a determinant of leaf structure and function among 23 amazonian tree species. Oecologia 86: 16-24.
